# Impact of an optimized surveillance protocol based on the European Association of Urology substratification on surveillance costs in patients with primary high-risk non-muscle-invasive bladder cancer

**DOI:** 10.1371/journal.pone.0275921

**Published:** 2023-02-10

**Authors:** Naoki Fujita, Shingo Hatakeyama, Kazutaka Okita, Masaki Momota, Yuki Tobisawa, Tohru Yoneyama, Hayato Yamamoto, Hiroyuki Ito, Takahiro Yoneyama, Yasuhiro Hashimoto, Kazuaki Yoshikawa, Chikara Ohyama

**Affiliations:** 1 Department of Urology, Hirosaki University Graduate School of Medicine, Hirosaki, Japan; 2 Department of Advanced Blood Purification Therapy, Hirosaki University Graduate School of Medicine, Hirosaki, Japan; 3 Department of Advanced Transplant and Regenerative Medicine, Hirosaki University Graduate School of Medicine, Hirosaki, Japan; 4 Department of Urology, Aomori Rosai Hospital, Hachinohe, Japan; 5 Department of Urology, Mutsu General Hospital, Mutsu, Japan; IRCCS Giovanni Paolo II Cancer Hospital, ITALY

## Abstract

**Objectives:**

The optimal frequency and duration of surveillance in patients with high-risk non-muscle-invasive bladder cancer (NMIBC) remain unclear. The aim of the present study is to develop an optimal surveillance protocol based on the European Association of Urology (EAU) substratification in order to improve surveillance costs after transurethral resection of bladder tumor (TURBT) in patients with primary high-risk NMIBC.

**Materials and methods:**

We retrospectively evaluated 428 patients with primary high-risk NMIBC who underwent TURBT from November 1993 to April 2019. Patients were substratified into the highest-risk and high-risk without highest-risk groups based on the EAU guidelines. An optimized surveillance protocol that enhances cost-effectiveness was then developed using real incidences of recurrence after TURBT. A recurrence detection rate ([number of patients with recurrence / number of patients with surveillance] × 100) of ≥ 1% during a certain period indicated that routine surveillance was necessary in this period. The 10-year total surveillance cost was compared between the EAU guidelines-based protocol and the optimized surveillance protocol developed herein.

**Results:**

Among the 428 patients with primary high-risk NMIBC, 97 (23%) were substratified into the highest-risk group. Patients in the highest-risk group had a significantly shorter recurrence-free survival than those in the high-risk without highest-risk group. The optimized surveillance protocol promoted a 40% reduction ($394,990) in the 10-year total surveillance cost compared to the EAU guidelines-based surveillance protocol.

**Conclusion:**

The optimized surveillance protocol based on the EAU substratification could potentially reduce over investigation during follow-up and improve surveillance costs after TURBT in patients with primary high-risk NMIBC.

## Introduction

Bladder cancer (BC) is the ninth most common cancer worldwide [1], with histological variants accounting for approximately 25% of newly diagnosed BCs [2]. Although 75% of patients with BC have non-muscle-invasive disease at diagnosis, the remaining 25% present with muscle-invasive, advanced, or metastatic disease [3]. Cisplatin-based chemotherapy remains the standard first-line treatment for patients with metastatic BC. Recently, immune checkpoint inhibitors have become novel treatment options [3, 4].

BC has been well-known as one of the most expensive cancers to manage on a per capita basis [5, 6]. Reports have shown that costs for managing patients with NMIBC have increased since 1993, driven by intravesical therapy and surveillance after transurethral resection of bladder tumor (TURBT) [7].

Several guidelines recommend risk-stratified surveillance protocols after TURBT in patients with NMIBC [8–10]. Accordingly, an intensive surveillance protocol has been recommended for high-risk NMIBC given its high recurrence and progression rates [11]. However, considering that no robust evidence exists [12–15], the optimal frequency and duration of surveillance remain unclear. In addition, although high-risk NMIBC is heterogenous and the European Association of Urology (EAU) guidelines suggest the substratification of the high-risk group into highest-risk and other groups [16], this is not reflected in their surveillance protocol [8]. Therefore, we speculated that an optimized surveillance protocol based on the EAU substratification might contribute toward reducing costs for patients with high-risk NMIBC.

The present study thus aimed to develop an optimal surveillance protocol based on the EAU substratification to improve surveillance costs after TURBT among patients with high-risk NMIBC.

## Materials and methods

### Ethics statement

This study was performed in accordance with the ethical standards of the Declaration of Helsinki and was approved by the Ethics Review Board of Mutsu General Hospital and Aomori Rosai Hospital (authorization numbers: H29-8 and 44, respectively). Pursuant to the provisions of the ethics committee and the ethics guidelines in Japan, retrospective and/or observational studies using existing documents do not require a written informed consent for the public disclosure of study information.

### Patient selection

A total of 480 patients with NMIBC who underwent TURBT from November 1993 to April 2019 at Mutsu General Hospital and Aomori Rosai Hospital were retrospectively evaluated. Among the 480 patients, 52 were excluded for satisfying one or more of the following exclusion criteria: (1) history of BC; (2) previous and/or simultaneous upper urinary tract (UUT) urothelial carcinoma (UC); (3) pure carcinoma in situ (CIS) of the bladder; and (4) low- or intermediate-risk in the EAU guidelines. Ultimately, 428 patients with primary high-risk NMIBC were included. Of these, 204 and 224 underwent TURBT at Mutsu General Hospital and Aomori Rosai Hospital, respectively.

### Evaluation of variables

The following variables were analyzed: age; sex; Eastern Cooperative Oncology Group performance status; body mass index; history of hypertension, diabetes mellitus, cardiovascular disease, and chronic kidney disease; number of tumors; tumor size; pathological T stage; tumor grade; variant histology of UC; lymphovascular invasion (LVI); postoperative intravesical instillation of chemotherapy and bacillus Calmette-Guérin (BCG); and second TURBT. Tumor stage was determined according to the 2009 TNM classification of the Union of International Cancer Control. Tumor grade was classified according to the 1973 World Health Organization classification system.

### Follow-up schedule

**Fig 1:**
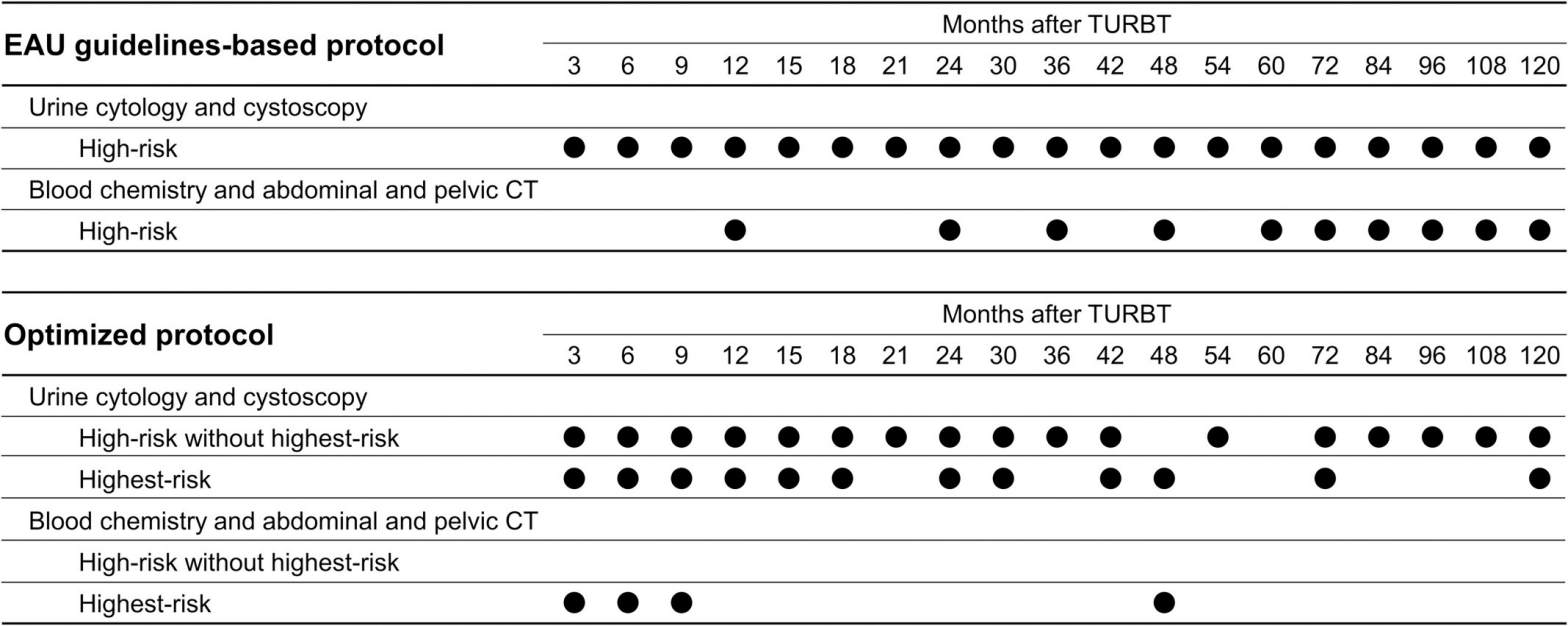
Surveillance protocols. EAU, European Association of Urology; TURBT, transurethral resection of bladder tumor; CT, computed tomography.

Our follow-up schedule was based on the EAU guidelines (urine cytology and cystoscopy every 3 months for 2 years, every 6 months for an additional 3 years, and annually thereafter; annual abdominal and pelvic computed tomography [CT], as well as serum chemistry screening to evaluate renal function for contrast-enhanced CT) (Fig 1, upper columns). Disease recurrence was classified as intravesical, UUT, and metastasis to any site, including local pelvic lymph nodes. The first recurrence site after TURBT was recorded.

### Optimized surveillance protocol

Based on the EAU 2019 guidelines [8], patients with pathological T1 (pT1) and grade 3 tumors associated with concurrent CIS of the bladder and/or the prostatic urethra, multiple and/or large pT1 and grade 3 tumors, some forms of variant histology of UC, and LVI were substratified into the highest-risk group. The optimized surveillance protocol that enhances cost-effectiveness was developed using real incidences of recurrence after TURBT. An intravesical recurrence detection rate ([number of patients with recurrence / number of patients with surveillance] × 100) of ≥ 1% during a certain period indicated that routine surveillance using urine cytology and cystoscopy was necessary in this period. On the other hand, an intravesical recurrence detection rate of < 1% during a certain period indicated that routine surveillance was not necessary in this period. Similarly, an UUT recurrence and/or metastasis detection rate of ≥ 1% during a certain period indicated that routine surveillance via CT and serum chemistry screening was necessary in this period. Based on these criteria, an optimized surveillance protocol was developed to improve surveillance costs.

### Outcome evaluation

Recurrence-free survival (RFS) (in any site), time to first recurrence in any site, estimated surveillance cost per one recurrence detection, and 10-year total surveillance cost using the EAU guidelines-based surveillance protocol and the optimized surveillance protocol were recorded. To estimate the cost-benefit, surveillance costs for detecting one recurrence were calculated (total surveillance cost in a follow-up period / number of patients with recurrence) using an exchange rate of 100 yen to one US dollar. Medical costs were $45 for urine cytology, $95 for cystoscopy, $267 for CT with contrast media, and $24 for blood chemistry. The cost breakdown was shown in S1 Table. The cost of prescriptions, medications, and doctor fees were not included herein. The total examination fee was calculated as the patients’ total fee (10%–30%) and national insurance coverage (70%–90%) in Japan. The 10-year total surveillance cost was compared between the EAU guidelines-based surveillance protocol and the optimized surveillance protocol developed herein.

### Statistical analysis

Statistical analyses were performed using SPSS version 24.0 (SPSS, Inc., Chicago, IL, USA) and GraphPad Prism 5.03 (GraphPad Software, San Diego, CA, USA). Categorical variables were compared using the Fisher exact test or chi-squared test. Quantitative variables were expressed as median with interquartile range. Differences between the two groups were compared using the Student *t* test and Mann–Whitney *U* test for normally and non-normally distributed data, respectively. The Kolmogorov–Smirnov test was used to determine the distribution of the data. RFS was evaluated using the Kaplan–Meier method and compared using the log-rank test. A *P* value of < 0.05 indicated statistical significance.

## Results

### Patient background

The median age and follow-up period after TURBT were 72 years and 52 months, respectively. Although 90 (21%) patients received postoperative intravesical instillation of BCG, no patient received maintenance BCG therapy. Among the 428 patients with primary high-risk NMIBC, 97 (23%) were substratified into the highest-risk group (Table 1). We obtained muscle specimens from all patients at the time of initial TURBT. No patient underwent early cystectomy.

**Table 1:** Patients’ background.

	All	High-risk without highest-risk	Highest-risk	*P* value
	n = 428	n = 331	n = 97	
Age, years	72 (64–79)	72 (63–79)	73 (67–78)	0.181
Male	342 (80%)	257 (78%)	85 (88%)	0.031
Body mass index, kg/m^2^	23 (21–25)	23 (21–25)	23 (21–26)	0.386
ECOG PS ≥1	59 (14%)	44 (13%)	15 (16%)	0.585
Hypertension	246 (58%)	185 (56%)	61 (63%)	0.220
Diabetes mellitus	72 (17%)	50 (15%)	22 (23%)	0.079
Cardiovascular disease	145 (34%)	104 (31%)	41 (42%)	0.047
Chronic kidney disease	140 (33%)	101 (31%)	39 (40%)	0.074
Multiple tumors	191 (45%)	120 (36%)	71 (73%)	<0.001
Tumor size				
≥ 3 cm	83 (19%)	48 (15%)	35 (36%)	<0.001
Pathological T stage				
pT1	415 (97%)	319 (96%)	96 (99%)	0.314
Concomitant carcinoma in situ	22 (5.1%)	16 (4.8%)	6 (6.2%)	0.596
Tumor grade				
Grade 3	125 (29%)	37 (11%)	88 (91%)	<0.001
Variant histology of urothelial carcinoma	10 (2.3%)	0 (0.0%)	10 (10%)	<0.001
Lymphovascular invasion	15 (3.5%)	0 (0.0%)	15 (16%)	<0.001
Intravesical instillation of chemotherapy	311 (73%)	236 (71%)	75 (77%)	0.242
Intravesical instillation of BCG	90 (21%)	63 (19%)	27 (28%)	0.061
Second TURBT	41 (9.6%)	25 (7.6%)	16 (17%)	0.009
First recurrence site				0.019
Intravesical	132 (95%)	98 (98%)	34 (87%)	
Upper urinary tract	5 (3.6%)	2 (2.0%)	3 (7.7%)	
Metastasis	2 (1.4%)	0 (0.0%)	2 (5.1%)	
Follow-up period, months	52 (26–93)	53 (26–95)	50 (26–86)	

All data is presented as n (%) or median (interquartile range). ECOG PS, Eastern Cooperative Oncology Group performance status; BCG, bacillus Calmette-Guérin; TURBT, transurethral resection of bladder tumor.

### Oncological outcomes

**Fig 2:**
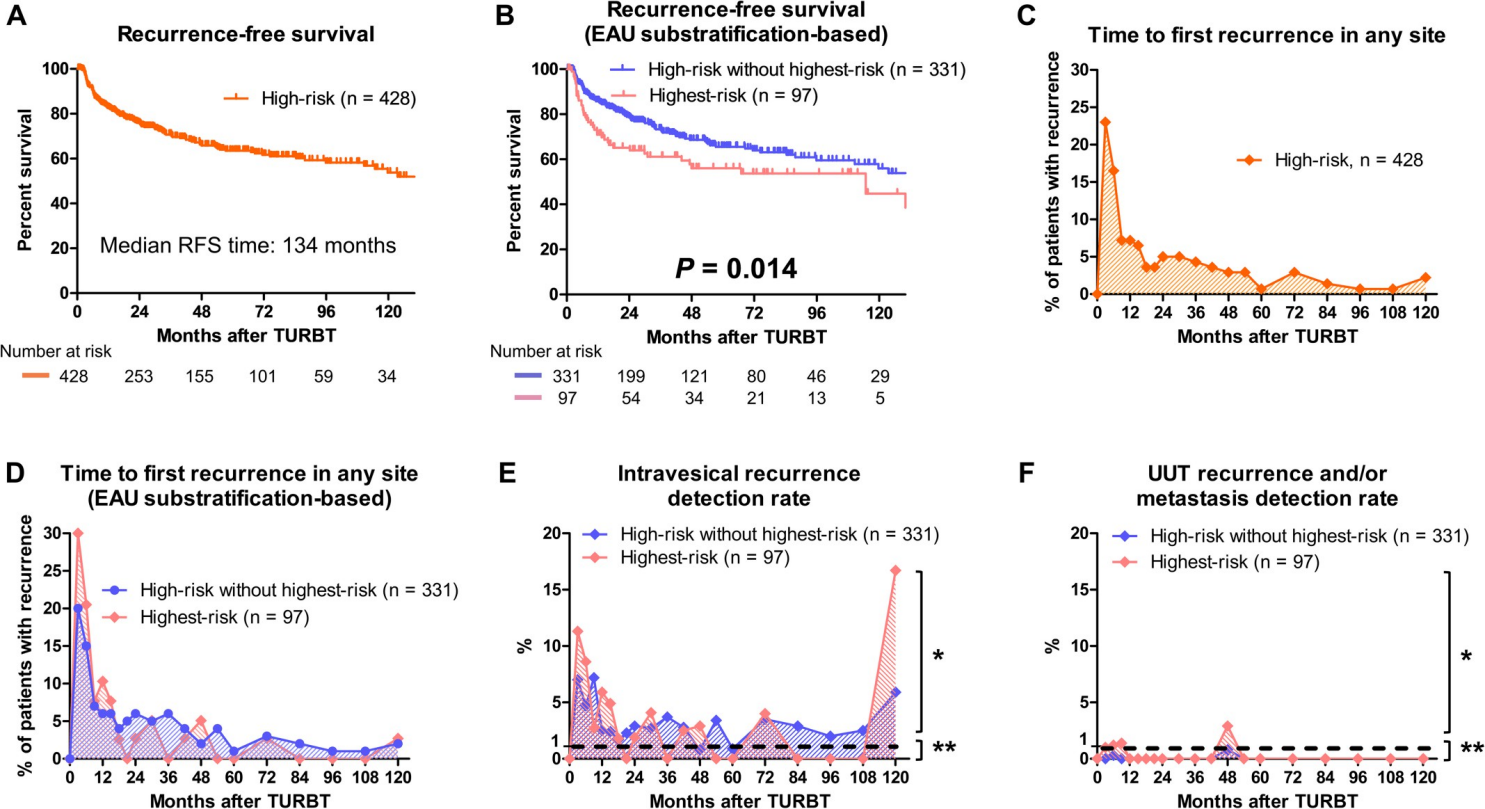
Recurrence-free survival (RFS), time to first recurrence in any site, and recurrence detection rate. RFS in all patients with high-risk non-muscle-invasive bladder cancer (NMIBC) was evaluated using the Kaplan–Meier method (**A**). RFS between the highest-risk and high-risk without highest-risk groups was compared using the log-rank test (**B**). Time to first recurrence in any site in patients with high-risk NMIBC (**C**) and highest-risk and high-risk without highest-risk NMIBC (**D**) were evaluated. Intravesical recurrence detection rate (**E**) and upper urinary tract (UUT) recurrence and/or metastasis detection rate (**F**) were evaluated. *, routine surveillance was needed (≥ 1%). **, routine surveillance was not needed (< 1%). TURBT, transurethral resection of bladder tumor. EAU, European Association of Urology.

All patients with high-risk NMIBC had a median RFS of 134 months (Fig 2A). Patients with highest-risk NMIBC had a significantly shorter RFS than those with high-risk without highest-risk NMIBC (Fig 2B; *P* = 0.014).

First recurrence sites were intravesical (n = 98, 98%) and UUT (n = 2, 2.0%) in patients with high-risk without highest-risk NMIBC, and intravesical (n = 34, 87%), UUT (n = 3, 7.7%), and metastasis (n = 2, 5.1%) in those with highest-risk NMIBC (Table 1). First recurrence occurred most frequently 3 months after TURBT in all patients with high-risk NMIBC (Fig 2C; n = 32, 23%) and gradually decreased thereafter, and patients with high-risk without highest-risk NMIBC had a similar time course for first recurrence (Fig 2D). Patients with highest-risk NMIBC had significantly more early recurrences within 1 year after TURBT than those with high-risk without highest-risk NMIBC (Fig 2D, 69% vs. 48%; *P* = 0.024). Only 2 (5.1%) recurrences occurred after 48 months in patients with highest-risk NMIBC (Fig 2D).

### Optimized surveillance protocol

Almost all patients with high-risk without highest-risk NMIBC had an intravesical recurrence detection rate of ≥ 1% throughout the follow-up period except at 48 and 60 months after TURBT (Fig 2E). On the other hand, patients with highest-risk NMIBC had an intravesical recurrence detection rate of < 1% at 21, 36, 54, 60, 84, 96, and 108 months (Fig 2E).

Patients with high-risk without highest-risk NMIBC had an UUT recurrence and/or metastasis detection rate of < 1% throughout the follow-up period (Fig 2F). On the other hand, patients with highest-risk NMIBC had an UUT recurrence and/or metastasis detection rate of ≥ 1% at 3, 6, 9, and 48 months (Fig 2F).

Based on previously discussed criteria (i.e., a recurrence detection rate of < 1% during a certain period indicated that routine surveillance was not necessary in this period), an optimized surveillance protocol was developed to improve surveillance costs (Fig 1, lower columns).

### Economic outcomes

**Fig 3:**
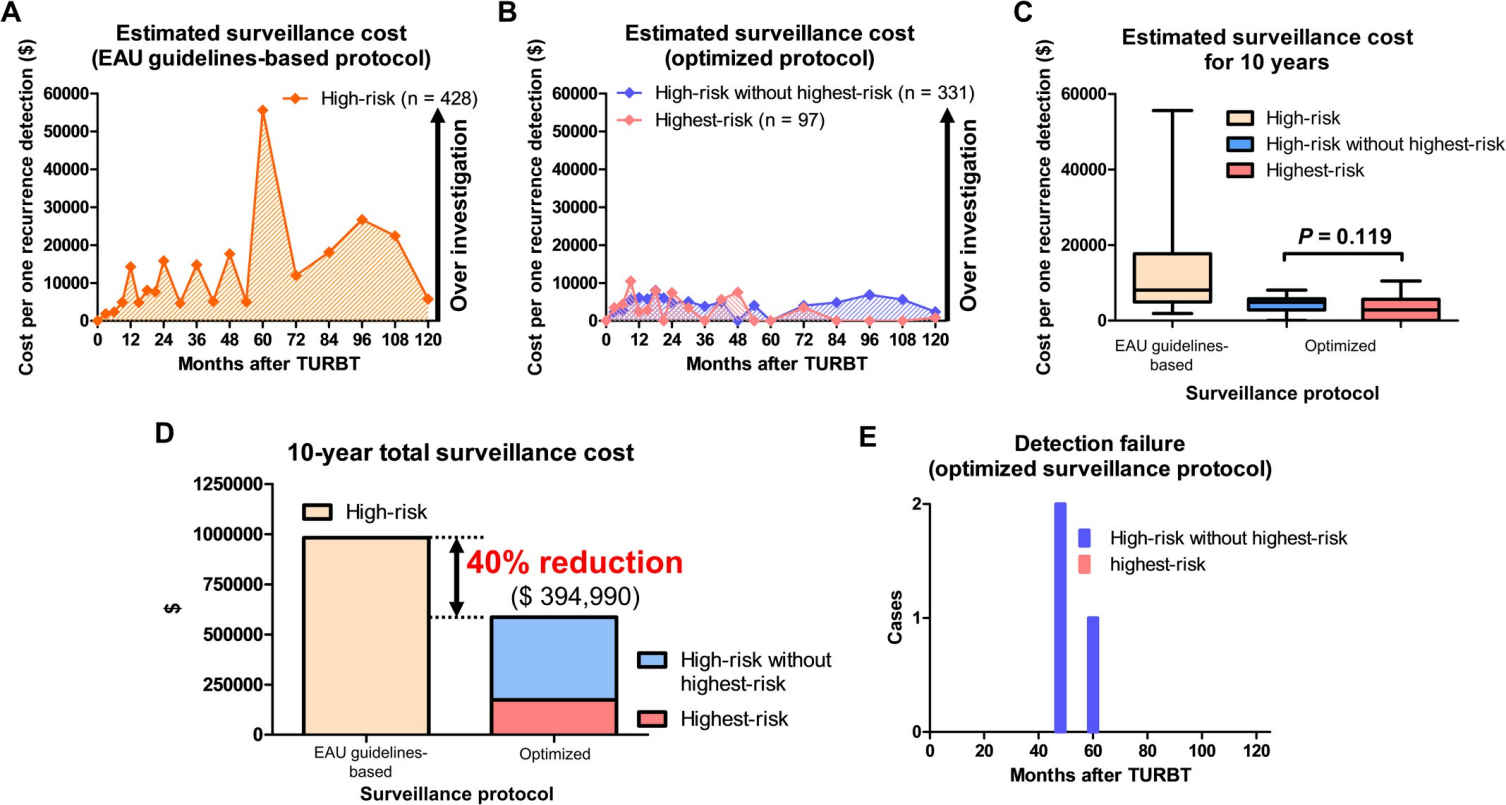
Estimated surveillance cost, 10-year total surveillance cost, and detection failure. Estimated surveillance costs per one recurrence detection (**A**: the European Association of Urology [EAU] guidelines-based surveillance protocol, **B**: the optimized surveillance protocol, and **C**; median costs) were evaluated. The optimized surveillance protocol promoted a 40% lower ($394,990) 10-year total surveillance cost compared to the EAU guidelines-based protocol (**D**). The number of patients that potentially failed recurrence detection using the optimized surveillance protocol (**E**). TURBT, transurethral resection of bladder tumor.

All patients with high-risk NMIBC had a higher (> $10,000) estimated surveillance cost per one recurrence detection at 12, 24, 36, 48, 60, 72, 84, 96, and 108 months (Fig 3A). On the other hand, patients with highest-risk and high-risk without highest-risk NMIBC had a lower (< $10,000) estimated surveillance cost per one recurrence detection throughout almost all of the follow-up period (Fig 3B).

Patients with high-risk NMIBC had a median surveillance cost of $8064 per one recurrence detection. No significant difference in median surveillance cost per one recurrence detection was observed between the highest-risk and high-risk without highest-risk groups (Fig 3C, $2847 vs. $4830; *P* = 0.119).

The optimized surveillance protocol promoted a 40% lower ($394,990) 10-year total surveillance cost compared to the EAU guidelines-based surveillance protocol (Fig 3D).

Only 3 (3.0%) patients with high-risk without highest-risk NMIBC and none of those with highest-risk NMIBC potentially failed recurrence detection using the optimized surveillance protocol (Fig 3E).

## Discussion

The optimal frequency and duration of surveillance in patients with primary high-risk NMIBC remain unclear. Although high-risk NMIBC is heterogenous and the EAU guidelines suggest the substratification of the high-risk group [16], the effect of this substratification on the cost-effectiveness also remains unclear. To the best of our knowledge, this has been the first study to evaluate cost-effectiveness of the optimized surveillance protocol based on the EAU substratification among patients with primary high-risk NMIBC. The present study showed that the optimized surveillance protocol promoted a 40% reduction in the 10-year total surveillance cost compared to the EAU guidelines-based surveillance protocol. The key points promoting cost reduction included the substratification of the high-risk group and the decrease in unnecessary UUT imaging, cystoscopy, and urine cytology. To reduce surveillance costs, cystoscopy and urine cytology after 48 months might be unnecessary for patients with highest-risk NMIBC due to low recurrence detection rates. Although we do not recommend our new surveillance protocol in clinical practice, these results may provide clinicians with the idea for a surveillance cost reduction. Considering the lack of evidence and increased costs for managing patients with NMIBC, further studies are warranted to develop an optimal surveillance protocol that balances oncological benefits with cost-effectiveness.

BC has been well-known as one of the most expensive cancers to manage on a per capita basis [5, 6]. Accordingly, reports have shown that the major drivers for increased cost included complications and surveillance among patients with BC [17, 18]. Moreover, Strope et al. reported that costs for managing patients with NMIBC have increased since 1993, driven by intravesical therapy and surveillance after TURBT [7]. Similarly, several studies have reported that majority of the costs were driven by cystoscopic surveillance [19, 20]. Accordingly, improving surveillance cost seems to be one of the most important factors for reducing total management costs of patients with NMIBC. Although several studies have reported possible surveillance strategies that improve surveillance costs using nuclear matrix protein 22, fibroblast growth factor receptor 3 mutation analysis, or fluorescence in situ hybridization assay in patients with NMIBC [21–23], such surveillance strategies remain impractical considering facility restrictions. Moreover, liquid biopsy is an alternative potential method for surveillance after TURBT [24]. Although liquid biopsy improves the sensitivity and specificity in recurrence detection, it may not be cost-effective for surveillance because of its high cost [24, 25]. Another strategy for improving surveillance costs involves the development of a surveillance protocol with optimal frequency and duration. Although the intensive surveillance protocol that is recommended for patients with high-risk NMIBC may detect recurrences before progression to muscle invasive BC [8–10], it promotes increased surveillance costs. Given the lack of evidence, an optimal surveillance protocol that enhances cost-effectiveness remains to be established. High-risk NMIBC is heterogenous and the EAU guidelines suggest the substratification of the high-risk group into the highest-risk and other groups [8, 16, 26]. Therefore, we speculated that an optimized surveillance protocol based on the EAU substratification might improve surveillance costs after TURBT. Our results showed apparent differences in the Kaplan–Meier curves for RFS between the highest-risk and high-risk without highest-risk groups. Moreover, the optimized surveillance protocol promoted a 40% lower 10-year total surveillance cost compared to the EAU guidelines-based surveillance protocol (Fig 3D). Although the heterogeneity of high-risk NMIBC makes developing an optimal surveillance protocol difficult, its results might be helpful for establishing a cost-effective surveillance protocol after TURBT in patients with primary high-risk NMIBC.

Although several guidelines recommend regular UUT imaging after TURBT in patients with high-risk NMIBC [8, 10], no robust evidence exists [12, 27, 28]. In addition, no study has demonstrated an association between regular UUT imaging and improved oncological outcomes. The present study revealed that the costs for regular abdominal and pelvic CT accounted for 43% of the 10-year total surveillance cost (S1 Fig). Moreover, patients with highest-risk NMIBC had an UUT recurrence detection rate of < 1% throughout the entire follow-up period except at 3, 6, 9, and 48 months after TURBT (Fig 2F). Although regular UUT imaging might detect asymptomatic UUT recurrences, no study has demonstrated the relationship between the detection of asymptomatic UUT recurrence and improved survival. Our additional analyses showed that asymptomatic recurrence was not associated longer cancer-specific survival and overall survival after UUT recurrence (S2A Fig, *P* = 0.911; S2B Fig, *P* = 0.555; respectively). Moreover, Sternberg et al. reported that of 3,074 CT examinations, only 15 (0.5%) could detect an UUT recurrence after TURBT [29]. These results suggest that the frequency and duration of regular UUT imaging might need to be reconsidered to balance oncological benefits with cost-effectiveness. Nonetheless, further studies on this matter are needed.

The present study developed an optimized surveillance protocol based on our original criteria (a recurrence detection rate of < 1% during a certain period indicated that routine surveillance was not necessary in this period). As such, patients with highest-risk NMIBC required less frequent routine surveillance using urine cytology and cystoscopy than patients with high-risk without highest-risk NMIBC. This result seems to be inconsistent with the theory that a more aggressive disease generally requires more intensive surveillance. However, 95% of patients with highest-risk NMIBC experienced intravesical recurrences within 48 months in the present study. Thus, less frequent surveillance might be sufficient after 48 months to improve cost-effectiveness considering that almost all recurrences in patients with highest-risk NMIBC occurred within 48 months after TURBT. Further studies are certainly needed to determine the optimal frequency and duration of surveillance among patients with highest-risk NMIBC.

The present study has several limitations worth noting. First, this study employed a retrospective design, which prevented us from making definitive conclusions. Second, a relatively small number of patients had been enrolled. Third, only 21% of the patients received intravesical instillation of BCG, none of whom received maintenance BCG therapy. In the study using the National Cancer Database that captured data on more than 70% of newly diagnosed cancer cases in the United States, Balakrishnan et al. have reported that of 47,694 patients with high-risk NMIBC, only 24% received BCG therapy regardless guideline recommendations [30]. Thus, the low BCG therapy rate in the present study may reflect a real-world clinical practice. Fourth, only a limited number of patients underwent second TURBT because it is difficult to perform second TURBT in all patients with high-risk NMIBC in clinical practice considering patients’ comorbidities and an operating room capacity. Fifth, the results obtained herein may not be generalized to other populations, given that the entire Japanese population is covered by universal health insurance (maximum copayment of 10% to 30%). Finally, the optimized surveillance protocol was developed based on our arbitrarily determined criteria. Thus, a validation study is needed.

## Conclusion

The optimized surveillance protocol based on the EAU substratification could potentially improve surveillance costs after TURBT in patients with primary high-risk NMIBC.

## Supporting information

S1 Fig Details on the 10-year total surveillance cost. Costs of regular abdominal and pelvic computed tomography for detecting upper urinary tract recurrence and/or metastasis accounted for 43% of the 10-year total surveillance cost.
(TIF)

S2 Fig Differences in oncological outcomes between patients with asymptomatic and symptomatic upper urinary tract (UUT) recurrences. Cancer-specific survival (**A**) and overall survival (**B**) were evaluated using the Kaplan–Meier method and compared using the log-rank test.
(TIF)

S1 Table Cost breakdown of urine cytology, cystoscopy, computed tomography, and blood chemistry test. (DOCX)
