# Supplementary figures and images for: Impact of an optimized surveillance protocol based on the European Association of Urology substratification on surveillance costs in patients with primary high-risk non-muscle-invasive bladder cancer
Source: PLoS One. 2023 Feb 10;18(2):e0275921. doi: 10.1371/journal.pone.0275921 (PMC9916549; doi:10.1371/journal.pone.0275921)

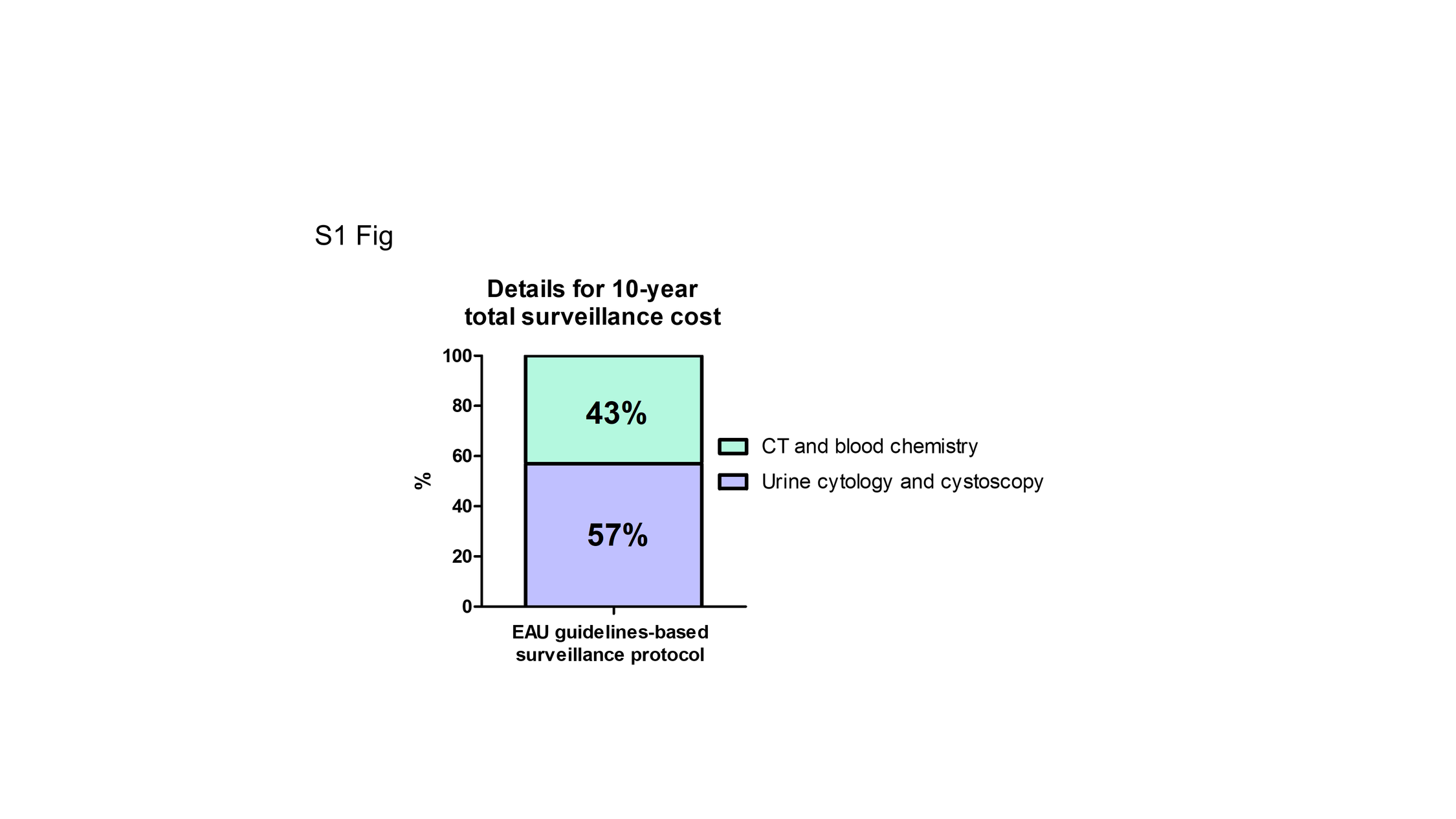

Supplement: S1 Fig — Costs of regular abdominal and pelvic computed tomography for detecting upper urinary tract recurrence and/or metastasis accounted for 43% of the 10-year total surveillance cost. (TIF) [file pone.0275921.s001.tif]

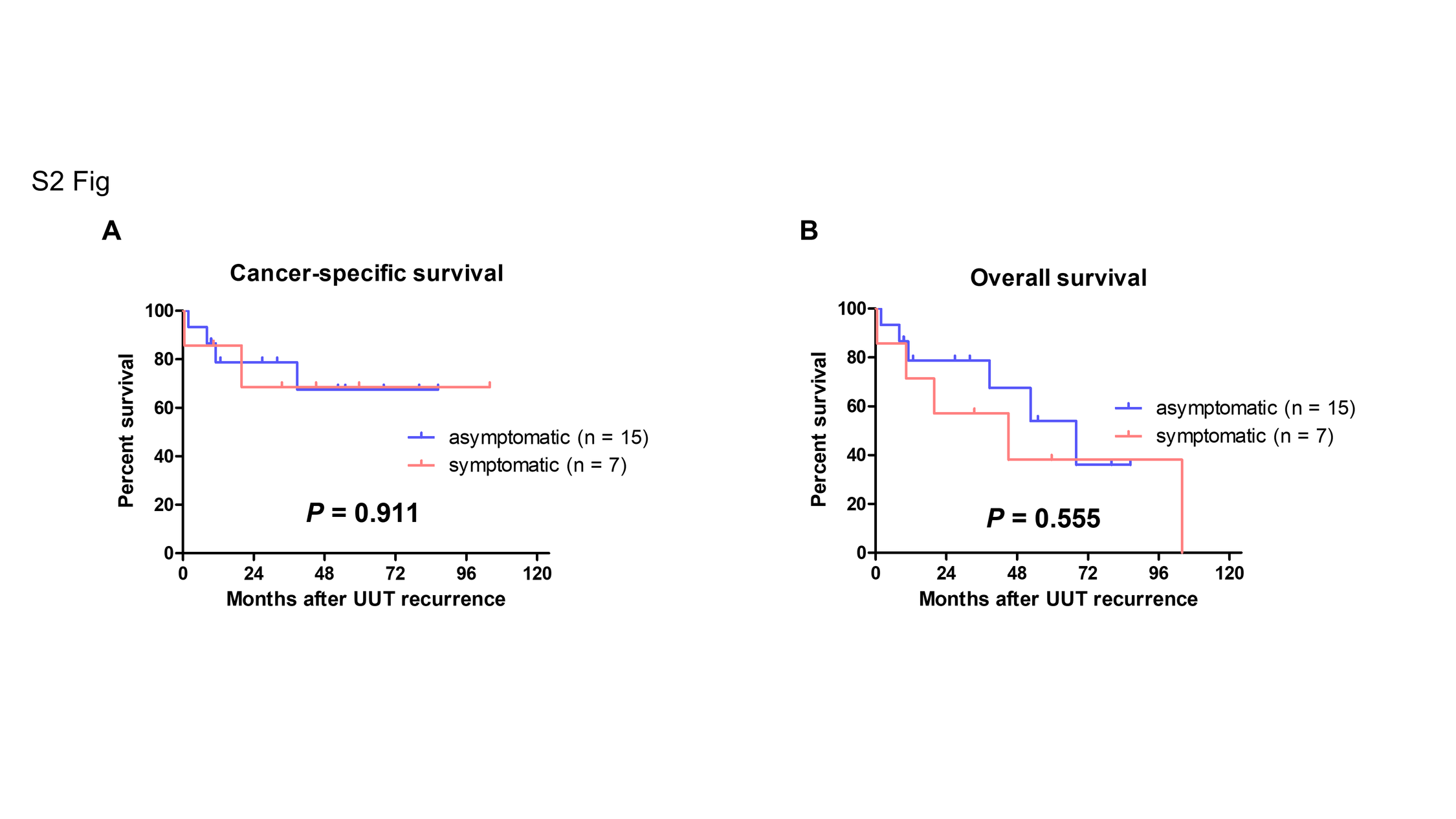

Supplement: S2 Fig — Cancer-specific survival (A) and overall survival (B) were evaluated using the Kaplan–Meier method and compared using the log-rank test. (TIF) [file pone.0275921.s002.tif]
