# Supplementary material for: Impact of an optimized surveillance protocol based on the European Association of Urology substratification on surveillance costs in patients with primary high-risk non-muscle-invasive bladder cancer
Source: PLoS One. 2023 Feb 10;18(2):e0275921. doi: 10.1371/journal.pone.0275921 (PMC9916549; doi:10.1371/journal.pone.0275921)
Supplement: S1 Table — (DOCX) [file pone.0275921.s003.docx]

**Supplementary Table 1 Cost breakdown of urine cytology, cystoscopy, computed tomography, and blood chemistry test**

| **Urine cytology** | **$45** |
| --- | --- |
| Examination fee | $19 |
| Diagnosis fee | $26 |
|  |  |
| **Cystoscopy** | **$95** |
| Examination fee | $95 |
|  |  |
| **Contrast-enhanced computed tomography** | **$267** |
| Scanning fee | $90 |
| Image diagnosis fee | $45 |
| Electronic image management fee | $12 |
| Contrast-media use fee | $50 |
| Contrast-media fee | $70 |
|  |  |
| **Blood chemistry test** | **$24** |
| Blood collection fee | $4 |
| Examination fee | $2 |
| Diagnosis fee | $14 |
| Blood sample management fee | $4 |
